# Metastatic gastroesophageal cancer in older patients – is this patient cohort represented in clinical trials?

**DOI:** 10.1186/s12885-021-09103-w

**Published:** 2022-01-03

**Authors:** Maeve A. Hennessy, Munzir Hamid, Niamh M. Keegan, Lynda Corrigan, Caitriona Goggin, Nay Myo Oo, Marie Carrigan, David Mockler, Anita O’Donovan, Anne M. Horgan

**Affiliations:** 1 Department of Medical Oncology, University Hospital Waterford, Waterford, Ireland; 2 St Lukes Radiation Oncology Oncology Network, St Lukes Rathgar, Dublin, Ireland; 3 John Stearne Library, Trinity Centre for Health Sciences, Dublin, Ireland; 4 Applied Radiation Therapy Trinity (ARTT), Trinity St James’s Cancer Institute, Trinity College, Dublin, Ireland

**Keywords:** Gastroesophageal, Metastatic, Phase III clinical trials

## Abstract

**Background:**

Older patients are underrepresented in the clinical trials that determine the standards of care for oncological treatment. We conducted a review to identify whether there have been age-restrictive inclusion criteria in clinical trials over the last twenty five years, focusing on patients with metastatic gastroesophageal cancer.

**Methods:**

A search strategy was developed encompassing Embase, PubMed and The Cochrane Library databases. Completed phase III randomised controlled trials evaluating systemic anti-cancer therapies in metastatic gastroesophageal malignancies from 1st January 1995 to 18th November 2020 were identified. These were screened for eligibility using reference management software (Covidence; Veritas Health Innovation Ltd). Data including age inclusion/exclusion criteria and median age of participants were recorded. The percentage of patients ≥ 65 enrolled was collected where available. The change over time in the proportion of studies using an upper age exclusion was estimated using a linear probability model.

**Results:**

Three hundred sixty-three phase III studies were identified and screened, with 66 trials remaining for final analysis. The majority of trials were Asian (48%; *n* = 32) and predominantly evaluated gastric malignancies, (86%; *n* = 56).

The median age of participants was 62 (range 18–94). Thirty-two percent (*n* = 21) of studies specified an upper age limit for inclusion and over half of these were Asian studies. The median age of exclusion was 75 (range 65–80). All studies prior to 2003 used an upper age exclusion (*n* = 12); whereas only 9 that started in 2003 or later did (17%). Among later studies, there was a very modest downward yearly-trend in the proportion of studies using an upper age exclusion (-0.02 per year; 95%CI -0.05 to 0.01; *p* = 0.31). Fifty-two percent (*n* = 34) of studies specified the proportion of their study population who were ≥ 65 years. Older patients represented only 36% of the trial populations in these studies (range 7–60%).

**Conclusions:**

Recent years have seen improvements in clinical trial protocols, with many no longer specifying restrictive age criteria. Reasons for poor representation of older patients are complex and ongoing efforts are needed to broaden eligibility criteria and prioritise the inclusion of older adults in clinical trials.

**Supplementary Information:**

The online version contains supplementary material available at 10.1186/s12885-021-09103-w.

## Background

Gastroesophageal cancers remain one of the most lethal malignancies, with 5 year survival rates of approximately 20–30% [1]. Typically these cancers affect older patients; 59% and 61% of patients with esophageal and gastric cancer respectively are 65 years or older at diagnosis [2]. Historically the older population has not been well-represented in the clinical trials that determine the standards of care for oncological treatment. The relative lack of representation of older patients in clinical trials is well documented with the proportion of participation reported to be as low as 25% [3, 4].

Cancers of the upper gastrointestinal tract have the propensity for early dissemination, and the majority of older patients present with locally-advanced, unresectable or metastatic disease [5].In general, combination chemotherapy with platinum doublet regimens have been shown to improve overall survival and provide higher response rates than single agents in the treatment of advanced gastric and esophageal cancer [6]. In the second-line setting, a survival benefit of chemotherapy over best supportive care has been demonstrated, although there is no consensus on the optimal regimen [6]. Although the highest incidence rates of advanced gastroesophageal cancers are among older patients, they are often treated with less intensive chemotherapy regimens, due to concerns regarding toxicity and tolerability, and this is in part due to the lack of evidence from phase III trials [5].

In more recent years, targeted therapies and immunotherapy have been evaluated in the treatment of advanced upper gastrointestinal malignancies. For example, for those with HER2 positive disease, the anti-HER2 directed monoclonal antibody trastuzumab has proven benefit [7]. The role of immunotherapy in the treatment of metastatic gastric and esophageal cancers continues to evolve, with new data showing significant survival benefits, challenging standard chemotherapy and targeted therapies in this setting [8]. These novel therapies are often more efficacious and less toxic than conventional cytotoxic chemotherapy and therefore show promise for an older population.

The number of older patients with advanced gastroesophageal cancer is expected to significantly increase globally due to the ageing population. Therefore it is paramount that we understand how best to treat this cohort of patients. Many clinical trials have imposed age inclusion criteria and therefore older patients are under-represented in large phase III clinical trials. The lack of clinical trial evidence for older patients has generated a significant challenge in translating trial results into clinical practice for a substantial proportion of patients.

Against this background, this study reviews all phase III trials of systemic therapy for advanced gastroesophageal cancer over the last 25 years. We aim to identify whether age restrictive criteria was specified in the trial protocols and thus to determine whether or not the current evidence base is applicable to the majority of patients diagnosed with this disease.

## Materials and methods

### Search strategy and study identification

A search strategy was developed by a health information specialist (M.C.) encompassing the following databases: Embase, PubMed and The Cochrane Library. The population of interest was patients with metastatic gastroesophageal malignancies and the intervention was systemic anti-cancer therapies. Medical Subject Headings (MeSH) terms and text words were identified for these components and linked using the AND operator. The search was filtered for phase III randomised clinical trials published between January 1st 1995 and November 18th 2020. Search terms were reviewed by A.H. and M.A.H. to ensure that the search strategy was comprehensive (for full details of all search terms, see Additional file 1). Additionally, we conducted a search of currently enrolling trials on clinicaltrials.gov to see if there has been any recent improvement in enrolment criteria.

### Selection criteria

The retrieved articles were imported into EndNote (version X9; Clarivate Analytics) and subsequently exported into a reference management software (Covidence; Veritas Health Innovation Ltd) for study selection. Two reviewers, (M.A.H., M.H.) independently screened study titles and abstracts for eligibility. Studies that were deemed eligible by title and abstract screening then underwent a full-text review by M.A.H. and M.H. using the same criteria. Any conflicts arising from this process were settled by discussion and with the help of a third reviewer (A.H.). Inclusion criteria were as follows: 1) English language; 2) full text available; 3) phase III randomised controlled trials; 4) trials evaluating outcomes for systemic therapies in advanced gastric, oesophageal or gastroesophageal cancer. Studies were excluded for the following reasons: 1) trials conducted in the neoadjuvant or adjuvant setting; 2) trials involving surgery or radiation; 3) no results published.

### Data extraction

The relevant information from eligible studies was extracted using a standardised template and this process was carried out by two reviewers (M.A.H. and M.H.). The following details were recorded: country of study, date of study onset, date of publication, patient number, age inclusion and exclusion criteria. In addition to the median age of the participants, the percentage of older patients, defined as age ≥ 65, enrolled in each study was collected where available. Where this information was not reported, it was sought from the corresponding authors via e-mail request. In cases where eligibility criteria were not directly available from the primary publication, the clinicaltrials.gov website was searched for this information.

### Statistical analysis

The change over time in the proportion of studies using an upper age exclusion was estimated using a linear probability model (i.e. linear regression with a binary outcome coded as [0,1]). The resulting model coefficients were reported with 95% confidence intervals and p-values, with p-values < 0.05 significant. All analyses were conducted using R (version 4.0.3). Figures were produced using ggplot2.

## Results

**Fig. 1:**
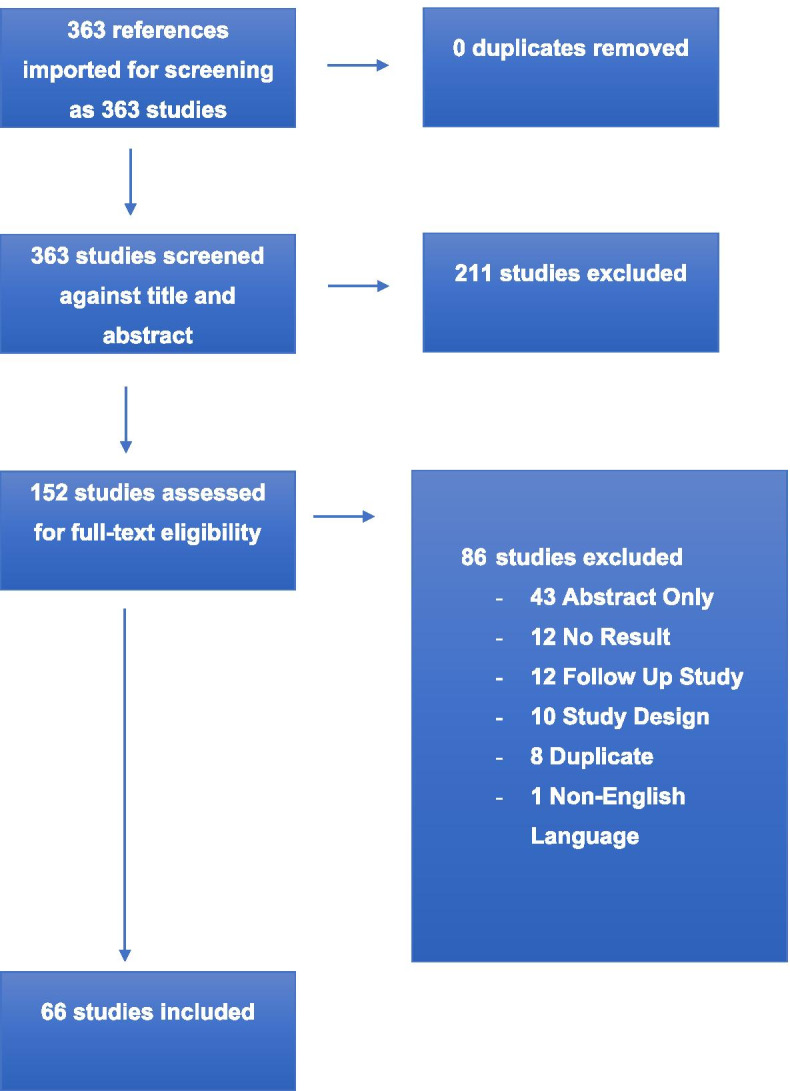
Preferred Reporting Items for Systematic Reviews and Meta-Analyses (PRISMA) Flow Diagram

A total of 363 phase III studies of systemic chemotherapy in advanced gastric, esophageal or gastroesophageal cancer were identified and screened for eligibility. One hundred and fifty two studies were eligible for full text review, 86 were excluded for reasons including duplication, no full text available, study design and non-English language, leaving 66 trials for final analysis (Fig. 1). Eighty six percent (*n* = 56) included gastric, 9% (*n* = 6) esophageal and 5% (*n* = 3) gastroesophageal malignancies. Asian studies represented 48% (*n* = 32) of trials included, 29% (*n* = 19) were worldwide and 23% (*n* = 15) were European (Table 1).

**Table 1:** Trial characteristics for included phase III studies of systemic anti-cancer therapy in advanced gastric, oesophageal or gastroesophageal cancer

	**n (Total *n* = 66)**	**%**
**Geographic Location**		
Asian	32	48
European	15	23
Worldwide	19	29
**Disease Site**		
Gastric	56	86
Gastroesophageal Junction	3	5
Oesophageal	6	9
**Line of Treatment**		
≥ 1	42	64
≥ 2	21	32
≥ 3	3	4
**Year of Publication**		
1995–2004	7	12
2005–2014	25	38
2015- to date	34	51
**Median Age of Participants**	62	(Range 18–94)
**Upper Age Restriction**		
Yes	21	32
No	43	65
Not Specified	2	3
**Median Cut Off Age**	75	(Range 65–80)

**Fig. 2:**
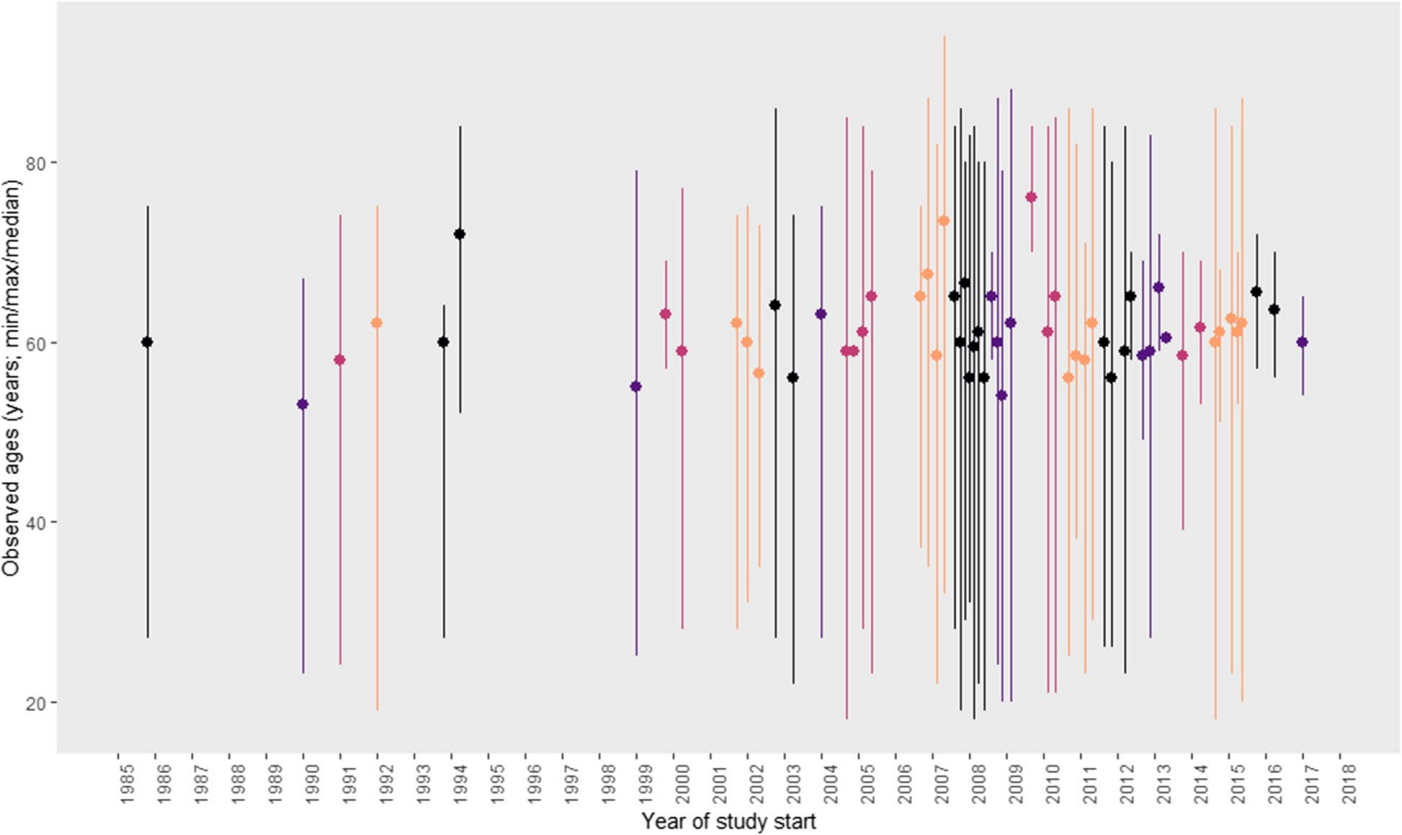
Observed ages of study participants (median and range) by year of study start (*n* = 66 studies; 1986 to 2017)

The median age of trial participants was 62, (range 18–94). There was no trend in the median age of trial participants over time (Fig. 2). Thirty two percent (*n* = 21) of studies specified an upper age limit for inclusion and 57% of these were Asian studies. Of the studies that specified an upper age limit for inclusion, the majority (*n* = 17; 81%), evaluated chemotherapy versus chemotherapy and most (*n* = 16; 76.2%), were in the first line metastatic setting. Two studies evaluated chemotherapy versus placebo (9.5%). One study (4.8%) looked at immunotherapy versus chemotherapy, for those who had at least two lines of prior therapy. One study (4.8%) evaluated targeted therapy versus best supportive care, for those who had received at least three lines of prior treatment in the metastatic setting. In the studies with an upper age limit, the median age of exclusion was 75 years (range 65–80).

**Fig. 3:**
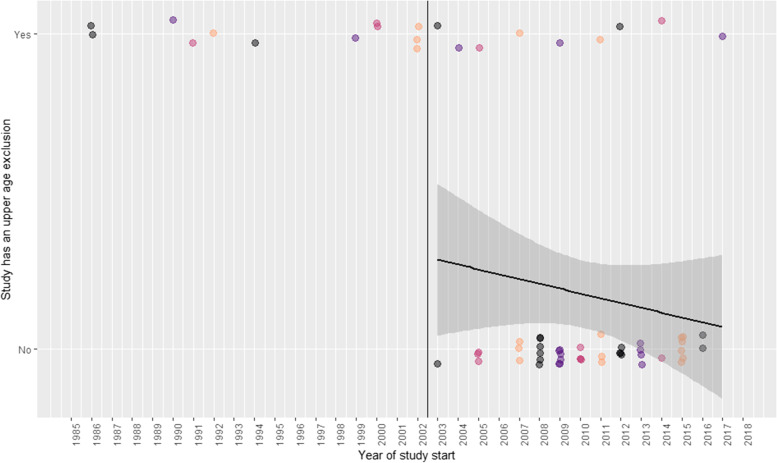
Numbers of studies with and without an upper age exclusion

All studies starting before 2003 used an upper age exclusion (*n* = 12); whereas only 9 of the 52 that started in 2003 or later did (17%). Among these later studies, there was a very modest downward yearly-trend in the proportion of studies using an upper age exclusion (-0.02 per year; 95%CI -0.05 to 0.01; *p* = 0.31) (Fig. 3).

Fifty-two percent (*n* = 34) of studies specified the proportion of their study population who were over 65 years. Older patients represented only 36% of the trial populations in these studies (range 7–60%). Recruitment of older patients did not appear to change over time. Of these, three studies also gave a further breakdown of patients over 70 and one trial specified the number of patients over 75 years. The outcomes reported were overall survival (OS) and progression free survival (PFS). Twenty-six (76%) of the 34 studies reported PFS/OS based on age in the subgroup analysis. Only one trial was specifically dedicated to older patients. This was a small Korean study (*n* = 50) published in 2014 which evaluated first-line chemotherapy with capecitabine monotherapy (x) versus capecitabine plus oxaliplatin (xelox) in elderly patients with advanced gastric cancer. Primary end point was to compare OS between the two randomly assigned arms (x vs. xelox). Secondary end points included PFS, response rate, safety and quality of life.

Our search of currently enrolling trials on clinicaltrials.gov yielded 11 active phase III studies in metastatic gastroesophageal cancer, none of which specified an upper age limit. All of these trials included chemotherapy with or without a combination of tyrosine kinase inhibitors, monoclonal antibodies or immunotherapy.

## Discussion

With an ageing population, where 60% of cancer diagnoses are made in patients over the age of 65, the inclusion of older patients in clinical trials is a priority [2, 9]. In our evaluation of phase III trials assessing systemic therapy for advanced gastroesophageal malignancies, 32% of the studies excluded patients based on older age alone. Furthermore, the median age of patients included was just 62, and this did not change over time. Those aged over 65 made up only 36% of the total study population. Consequently, it is difficult to apply this evidence to our everyday clinical practice, where we frequently encounter older, frailer and more complex patients than the individuals included in these trials. This may lead to both suboptimal treatment of some ‘fitter’ patients and over-treatment of those who may be more frail than their biological age, resulting in detrimental patient outcomes.

Reasons for poor representation of older adults in clinical trials are complex and multifactorial. They relate to a mix of patient, physician and system factors. There have been a few studies examining patient perspectives and attitudes towards clinical trial participation. Townsley et al. conducted a study focusing on understanding the attitudes of elderly patients with cancer towards clinical trial enrolment [10]. Over 80% of respondents were between the ages of 70 and 79 and the majority of patients stated they would participate in clinical trials to prevent or screen for cancer, to compare a new drug to a 'standard' drug, and 70% would participate in clinical trials to test a new drug in situations where there is no 'standard' drug [10]. However, while most were willing to consider participation when offered, few elderly patients actively sought clinical trials and overall were less well informed regarding the availability of relevant clinical trials [10]. A study by Yellen et al. looked at age and clinical decision-making in oncology patients using clinical vignettes in an interview situation. They found that older patients were as likely as the younger cohort to agree to chemotherapy for both curative and disease control purposes [11]. Ayodele et al. carried out a similar study to compare the attitudes of younger and older patients to clinical trials and found that older patients were as willing as younger patients to participate in clinical trials, yet significantly less were enrolled [12]. In reality, the inclusion of older patients is not always straightforward and may not be feasible, due to comorbidities, cognitive issues or social circumstances. Multiple clinic visits, paperwork and travel to medical appointments are well-documented barriers to trial accrual, and reducing trial participation burden is an area where further progress is needed [9].

Misconceptions among physicians can also act as a barrier to trial enrollment. In a survey of American oncologists, 50% indicated that they declare patients unsuitable for clinical trials based on age alone [13]. Another study examining barriers to clinical trial participation in older women with breast cancer, found that the physicians’ perceptions about age and tolerance of toxicity were the greatest obstacle to enrolling older women onto trials [14]. Sedrek et al. carried out semi-structured interviews with 44 medical oncologists (24 academic-based and 20 community-based) in an attempt to explore oncologists’ perceptions of barriers to clinical trial enrollment of older adults with cancer. The most common barriers identified by oncologists were stringent eligibility criteria and concerns for treatment toxicities [15]. A better awareness of clinical trials must be promoted amongst healthcare providers in order to increase older adult participation.

Efforts to increase the representation of older adults has been recognized as a priority by a number of international oncological organizations. The International Society of Geriatric Oncology (SIOG) published updated guidance in January 2021 relating to ‘Priorities for global advancement of care for older adults with cancer’ [16]. This policy document discusses priorities relating to education, clinical practice, research, and collaborations in an attempt to improve healthcare for this rapidly growing patient cohort.

In February 2021, the American Society of Clinical Oncology (ASCO) and the Friends of Cancer Research (Friends) group issued new recommendations to further broaden eligibility criteria for clinical trials, with the aim of expanding patient access [17]. Although the underlying rationale for eligibility criteria is to protect the safety of trial participants and to exclude patients who may have an unacceptably high risk of toxicity, this must be balanced with need to include a representative group of individuals [17, 18]. Interestingly, an analysis conducted from CancerLinQ Discovery® (CancerLinQ’s deidentified real-world data product for researchers) found that the number of lung cancer patients potentially suitable for clinical trials almost doubled, when three common eligibility criteria (renal function, presence of brain metastases, history of prior malignancy) were relaxed [19].

In addition to the broadening of eligibility criteria, other suggestions to promote inclusion of older patients in trials focus on addressing the study design, statistical analysis and reporting of trial results. Our search demonstrated that although the traditional outcomes of PFS and OS were evaluated in these phase III trials, more ‘age-relevant’ endpoints such as functional status and quality of life were not assessed. Similarly, expanding treatment-related toxicities to include adverse effects relevant to elderly patients, such as incontinence and falls has been recommended [9, 20, 21]. In terms of reporting of results, it is advised that age-specific subgroup analyses should be powered to detect any age related differences, and in situations where sub-group analysis is not pre-specified, any conclusions should be described as exploratory [22]. Tackling the above issues will help to strengthen and develop our evidence base and allow better decision making for complex, older patients. In our study, we noted that all studies that specified the proportion of patients > 70 years were published after 2014, which perhaps highlights better awareness in more recent years. Additionally it is a sign that the cut-off of 65 years which has traditionally been used to define an ‘older’ patient, is evolving over time.

Clinical trials can also specifically focus on older adults with cancer and indeed there have been a number of ‘elderly-specific’ trials in the last few years, highlighting that it is possible to conduct phase III trials in this patient population [23–28]. In the context of gastroesophageal cancer, the GO2 trial was a large phase III study which included 514 older patients with advanced gastroesophageal cancer who were unfit for full dose chemotherapy and aimed to find the optimal dosing strategy. Patients were randomized (1:1:1) to oxaliplatin and capecitabine (xelox) on 3 different dose schedules. The lowest dose demonstrated decreased rates of toxicity and improved quality of life, without shortening survival [28]. These studies illustrate that large randomized studies on older patients are feasible and contribute to creating a body of evidence that guides clinical decision making in this setting.

Overall, there is a slow but definite shift towards including older and multimorbid patients in clinical trials, and certainly the creation of ‘elderly-specific’ trials as mentioned above is important and encouraging. Interestingly, in our study, when we analyzed the patterns of enrollment over the time, we found that age limits were much more common pre-2003. Perhaps this was influenced by the publication by Hutchins et al. in the New England Journal of Medicine in 1999, ‘Underrepresentation of patients ≥ 65 in cancer treatment trials.’ In this study, data on 16,396 patients enrolled in clinical trials between 1993 and 1996 particularly focusing on sex, race and age, were analyzed and compared with rates of cancer in the general population, according to the US Census and the National Cancer Institute [4]. Efforts had been made previously to address the under-representation of women and minority ethnic groups, and the overall proportions of these cohorts were found to be similar. In contrast, patients 65 years or older were dramatically under represented (25% versus 63%, *p* < 0.001) [4]. Since then a number of policies have been developed in an attempt to remedy this. As part of our study, we accessed clinicaltrials.gov and we found that none of the currently active and enrolling phase III clinical trials in advanced gastroesophageal cancer specified an upper age limit for inclusion. It is encouraging that trials have reduced the explicit upper age limits and now we must move to focusing on the other barriers that disproportionately exclude older individuals. In the interim, there is a value to using observational data to study treatment effects in older patients with cancer [29–31]. These studies can provide information on frailer, co-morbid patients, those that are more representative of patients seen in daily practice. Notwithstanding the fact that selection biases may impact the validity of using observational data to estimate benefits of therapies, these can complement the results from randomized controlled trials in which patients are highly selected.

This study provides important information regarding the under-representation of older adults in clinical trials. We focused on analysis of age inclusion criteria, however we acknowledge that there are multiple other factors that can contribute to the low accrual of older adults in clinical trials. These include co-morbidities, renal function, liver function, cognitive and functional status and were beyond the scope of this study. Older patients are a heterogenous cohort, with varying levels of functional status and comorbidities. It is important to consider that strict inclusion criteria mean that the subjects enrolled in clinical trials, even in the oldest cohort, often don’t represent older patients in the general population [3, 32].

In conclusion, recent years have seen improvements in clinical trial protocols, with many no longer specifying restrictive age criteria. With an ageing population there is a growing need to include older, frailer patients who are more reflective of the ‘real world’ oncology patient in clinical trials. Progress has certainly been made, and we must continue to advocate for an inclusive culture and strive to generate the best evidence that will allow us to make informed treatment decisions for our patients.

## Supplementary Information

**Additional file 1.** Appendix.

## Data Availability

The datasets used and/or analysed during the current study are available from the corresponding author on reasonable request.

## Abbreviations

MeSH: Medical Subject Headings
SIOG: International Society of Geriatric Oncology
ASCO: American Society of Clinical Oncology
PFS: Progression Free Survival
OS: Overall Survival
