# Supplementary material for: Metastatic gastroesophageal cancer in older patients – is this patient cohort represented in clinical trials?
Source: BMC Cancer. 2022 Jan 3;22:3. doi: 10.1186/s12885-021-09103-w (PMC8722002; doi:10.1186/s12885-021-09103-w)
Supplement: Supplementary file 1 — Additional file 1. Appendix. [file 12885_2021_9103_MOESM1_ESM.docx]

Appendix

Search Strategy:

(((((ESOPHAG*[TITLE/ABSTRACT] OR OESOPHAG*[TITLE/ABSTRACT] OR GASTROESOPHAG*[TITLE/ABSTRACT] OR GASTROOESOPHAG*[TITLE/ABSTRACT] OR GASTRIC[TITLE/ABSTRACT]) AND (NEOPLAS*[TITLE/ABSTRACT] OR CANCER*[TITLE/ABSTRACT] OR TUMO*[TITLE/ABSTRACT] OR CARCINOMA[TITLE/ABSTRACT])) OR ("ESOPHAGEAL NEOPLASMS"[MESH])) AND (METASTA*[TITLE/ABSTRACT] OR SECONDAR*[TITLE/ABSTRACT] OR SPREAD[TITLE/ABSTRACT] OR ADVANCED[TITLE/ABSTRACT])) AND ((CHEMOTHERAP*[TITLE/ABSTRACT] OR CHEMORADI*[TITLE/ABSTRACT] OR RADIO CHEMO[TITLE/ABSTRACT]) OR ("DRUG THERAPY"[MESH]))) AND (RANDOM*[TITLE/ABSTRACT] OR PLACEBO*[TITLE/ABSTRACT] OR SINGLE BLIND*[TITLE/ABSTRACT] OR DOUBLE BLIND*[TITLE/ABSTRACT] OR TRIPLE BLIND*[TITLE/ABSTRACT]) FILTERS: CLINICAL TRIAL, PHASE III
